# Isolation and functional characterization of two dioxygenases putatively involved in bixin biosynthesis in annatto (*Bixa orellana* L.)

**DOI:** 10.7717/peerj.7064

**Published:** 2019-06-21

**Authors:** Victor Manuel Carballo-Uicab, Yair Cárdenas-Conejo, Alba Adriana Vallejo-Cardona, Margarita Aguilar-Espinosa, Jacobo Rodríguez-Campos, Hugo Serrano-Posada, José Alberto Narváez-Zapata, Felipe Vázquez-Flota, Renata Rivera-Madrid

**Affiliations:** 1 Unidad de Bioquímica y Biología Molecular de Plantas, Centro de Investigación Científica de Yucatán A.C., Mérida, Yucatán, México; 2 Laboratorio de Agrobiotecnología. CONACYT, Universidad de Colima, Colima, Colima, México; 3 Unidad de Biotecnología Médica y Farmacéutica, CONACYT, Centro de Investigación y Asistencia en Tecnología y Diseño del Estado de Jalisco, Guadalajara, Jalisco, México; 4 Unidad de Servicios Analíticos y Metrológicos, Centro de Investigación y Asistencia en Tecnología y Diseño del Estado de Jalisco, Guadalajara, Jalisco, México; 5 Instituto Politécnico Nacional-Centro de Biotecnología Genómica, Taumalipas, Tampico, México

**Keywords:** Annatto, Carotenoid dioxygenase, Bixin synthesis, *Bixa orellana*, Apocarotenoids, Bixin aldehyde

## Abstract

Carotenoid cleavage dioxygenases (CCDs) are enzymes that have been implicated in the biosynthesis of a wide diversity of secondary metabolites with important economic value, including bixin. Bixin is the second most used pigment in the world’s food industry worldwide, and its main source is the aril of achiote (*Bixa orellana* L.) seeds. A recent transcriptome analysis of *B. orellana* identified a new set of eight CCD members (BoCCD4s and BoCCD1s) potentially involved in bixin synthesis. We used several approaches in order to discriminate the best candidates with CCDs genes. A reverse transcription-PCR (RT-qPCR) expression analysis was carried out in five developmental stages of two accessions of *B. orellana* seeds with different bixin contents: (P13W, low bixin producer and N4P, high bixin producer). The results showed that three BoCCDs (BoCCD4-1, BoCCD4-3, and BoCCD1-1) had an expression pattern consistent with bixin accumulation during seed development. Additionally, an alignment of the CCD enzyme family and homology models of proteins were generated to verify whether the newly proposed CCD enzymes were bona fide CCDs. The study confirmed that these three enzymes were well-preserved and belonged to the CCD family. In a second selection round, the three CCD genes were analyzed by in situ RT-qPCR in seed tissue. Results indicated that BoCCD4-3 and BoCCD1-1 exhibited tissue-specific expressions in the seed aril. To test whether the two selected CCDs had enzymatic activity, they were expressed in *Escherichia coli*; activity was determined by identifying their products in the crude extract using UHPLC-ESI-QTOF-MS/MS. The cleavage product (bixin aldehyde) was also analyzed by Fourier transform infrared. The results indicated that both BoCCD4-3 and BoCCD1-1 cleave lycopene in vitro at 5,6-5′,6′.

## Introduction

**Figure 1:**
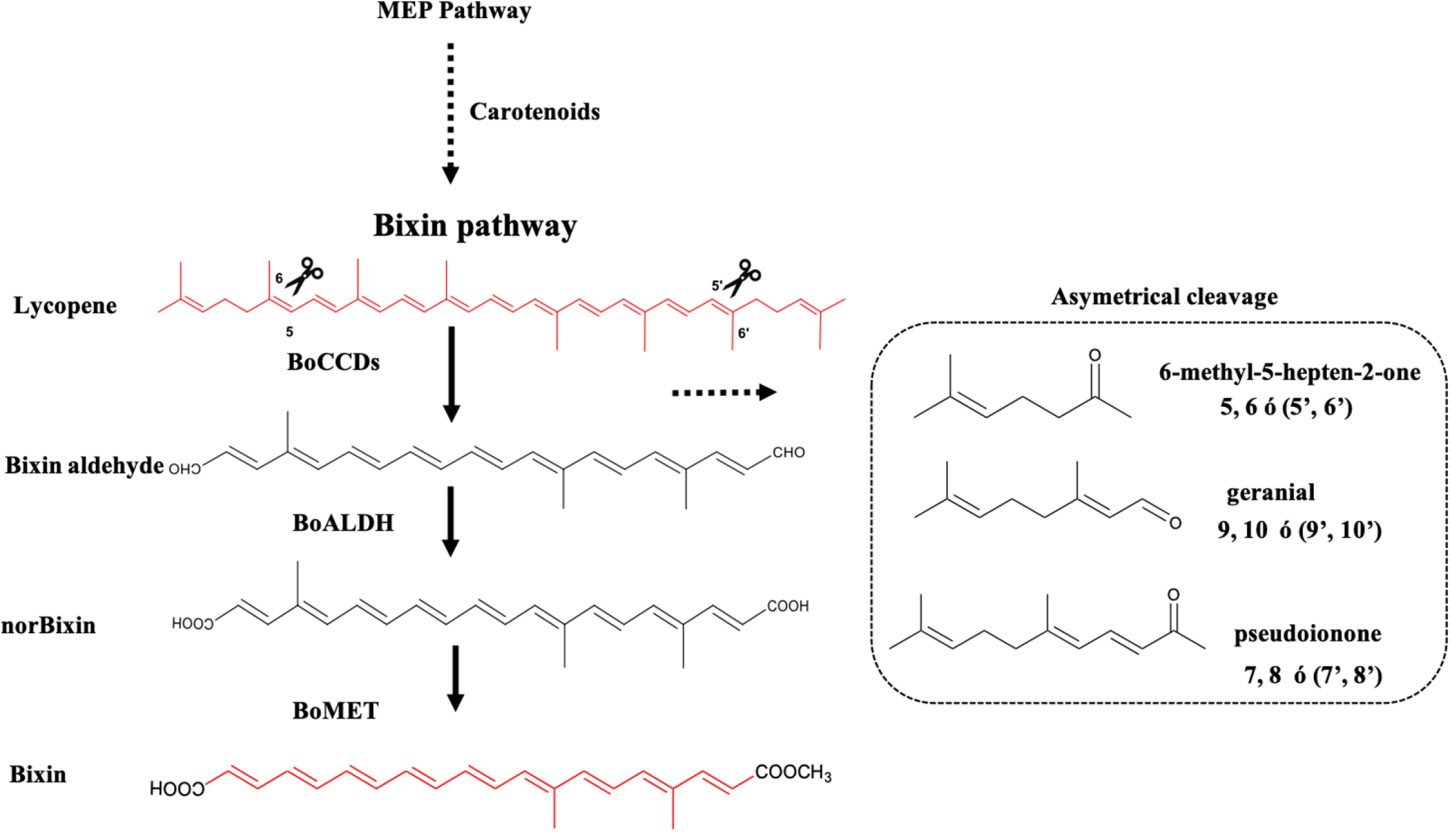
Bixin biosynthesis in *B. orellana* L. The possible cleavage sites and BoCCDs involved were determined based on the substrates and cleavage sites of type 4 CCDs. The region selectivity has been well-characterized in this and other plants. BoCCD, Carotenoid cleavage dioxygenases; BoALDH, bixin aldehyde dioxygenase; BoMET, norbixin methyltransferase.

The seeds of the achiote (or annatto-tree) (*Bixa orellana*) are the sole commercial source of bixin, a red orange colorant widely used in the food industry (Rivera-Madrid et al., 2016). Bixin belongs to the apocarotenoid pigment group, which are formed from the oxidative breakdown of carotenoids via the action specific carotenoid cleavage enzymes (CCE). CCE’s are members of the polyene chain oxygenases superfamily, a group of enzymes distributed across all taxa and classified according to their substrates and the position of the scissile bond (Ryle & Hausinger, 2002). In plants, two types of carotenoid dioxygenases have been identified, which differ in their cleavage activity. One type includes the 9-cis-epoxy-carotenoid cleavage dioxygenases, which act simultaneously on both the 11, 12, and 11′,12′ double-bonds of 9-cis-epoxycarotenoids, such as in violaxanthin and neoxanthin. This group is represented by the maize ABA-deficient VP14 enzyme, catalyzing the first step in ABA biosynthesis represents this group (Schwartz et al., 1997). The second group is composed of carotenoid cleavage dioxygenases (CCDs), which can cleave either or both of the double bonds. Furthermore, whereas some CCD enzymes could display a strict specificity toward their carotenoid or apocarotenoid substrates, others show a wider preference to different substrates. CCD enzymes are grouped into in five classes: CCD1, CCD2, CCD4, CCD7, and CCD8 (Auldridge, McCarty & Klee, 2006; Frusciante et al., 2014; Walter & Strack, 2011). Previously, a CCD4-type *B. orellana* dioxygenase catalyzing the simultaneous oxidation of the 5,6 and (5′,6′) double bonds of lycopene (BoLCD) was identified in a *B. orellana* sample collected in Abidjan (Ivory Coast Africa) (Bouvier, Dogbo & Camara, 2003). This enzyme is proposed to participate in the initial reaction of bixin biosynthesis, rending the bixin aldehyde, which after being oxidized to norbixin is methylated to produce bixin. The corresponding bixin aldehyde dehydrogenase and norbixin methyltransferase involved in theses reactions were also presumably identified (Bouvier, Dogbo & Camara, 2003). However, in a recent analysis of a photosynthetic (leaf) and nonphotosynthetic (immature and mature seed) transcriptome from a bixin-producing *B. orellana* Yucatecan accession, none of the genes putatively involved in the process were found (Cárdenas-Conejo et al., 2015; Fig. 1). Moreover, different research groups also have failed to detect the proposed bixin biosynthetic genes using different plant genotypes (Rodríguez-Ávila et al., 2011b; Sergeant et al., 2009; Walter & Strack, 2011). Interestingly, a number of sequences related to those presumably involved in bixin biosynthesis were identified in the transcriptome obtained from the Yucatecan cultivar (Cárdenas-Conejo et al., 2015; Rivera-Madrid et al., 2016). Remarkably, two upregulated CCD-1 and -4 enzymes, named as *BoCCD1-1* and *BoCCD4-3*, were detected in immature seeds and coincided with bixin accumulation (Cárdenas-Conejo et al., 2015). The concurrence of the time and space accumulation of bixin with the increased expression of these genes suggests their possible involvement in the process. Therefore, the aim of this research was to identify plausible BoCCDs enzymes involved in bixin synthesis. To this end, different approaches were implemented, including a comparison of the expression levels of *BoCCDs* candidates in *B. orellana* accessions that differ in their bixin accumulation and tissue transcript localization. Additionally, homology modeling and functional analysis through the expression of the genes in *Escherichia coli* was performed.

The expression of both *BoCCD1-1* and *BoCCD4-3* was markedly higher in the selected bixin producer, the N4P accession, in comparison with the low producer, P13W. Moreover, gene expression followed the same profile as bixin accumulation through seed development and was exclusively located in the seed aril, which is the site of bixin formation. Finally, *E. coli* cells expressing both *BoCCDs* acquired the capacity to produce bixin aldehyde from lycopene, confirming their 5,6-5′,6′cleavage activity. Taken together, these data suggest the involvement of these new dioxygenases in bixin biosynthesis.

## Materials and Methods

### Plant material

**Figure 2:**
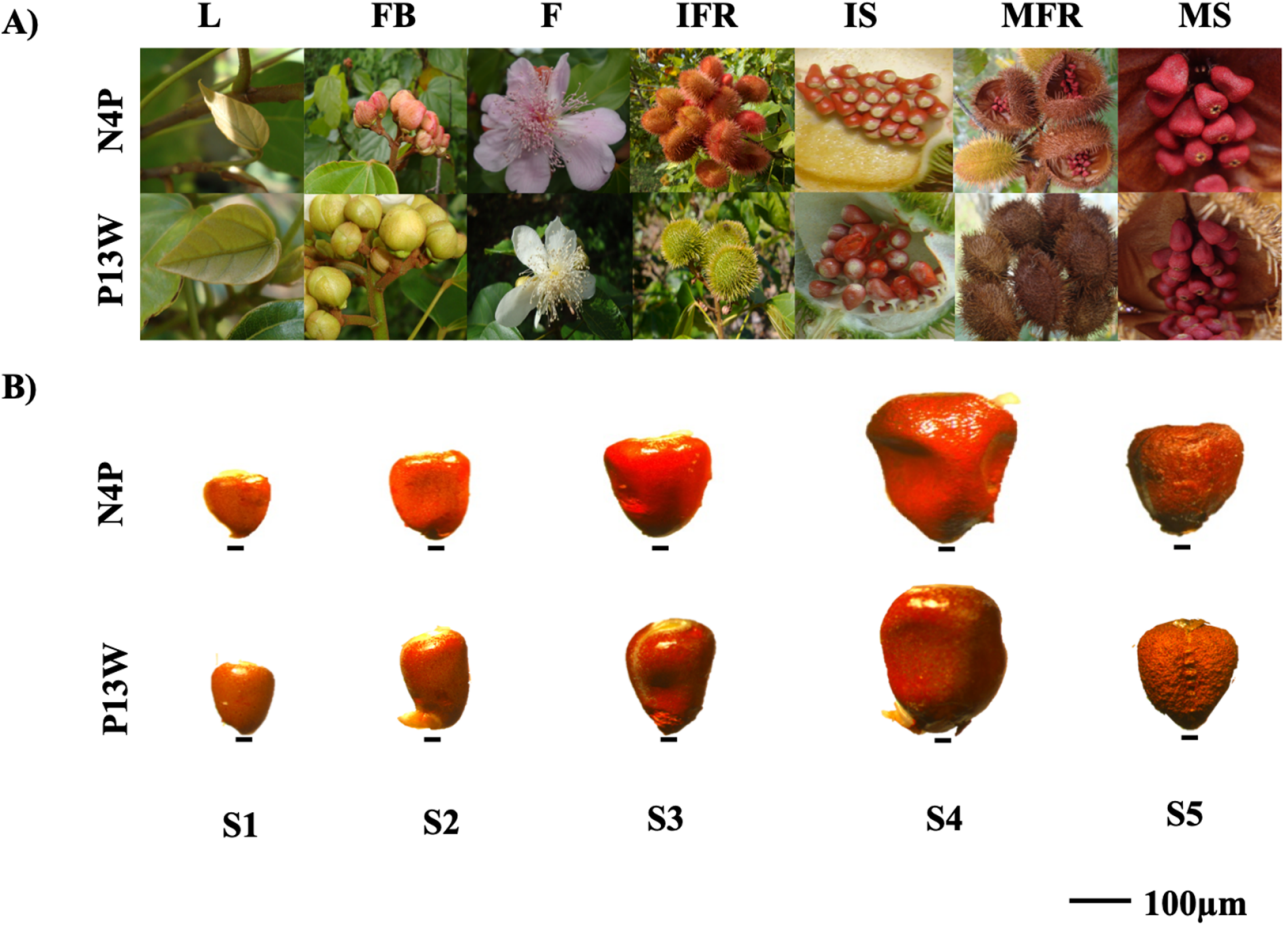
Annatto cultivars with contrasting morphological characteristics. (A) N4P and P13W cultivar characteristics. L, leaf; FB, floral bud; F, flower; IFR, immature fruit; IS, immature seed; MFR, mature fruit; MS: mature seed. (B) General representation of seeds in *B. orellana* accessions. Stages of seed development: S1–S5. Bar. 100 μm. Photos by Victor Manuel Carballo-Uicab.

Two achiote accessions N4P and P13W differing in their bixin contents and morphological characteristics, were selected for this study. Accession N4P presents pink flowers, red dehiscent capsules (fruits), and high bixin content (16.04 ± 0.52c mg/g DW). Accession P13W displays white flowers, green indehiscent capsules, and low bixin contents (08.76 ± 0.79a mg/g DW) (Trujillo-Hdz et al., 2016) (Fig. 2A). These accessions are part of a regional *B. orellana*. germplasm collection located at Temozón Norte (Mérida District) Yucatán, México (21°03′52″/89°35′48″). Seeds were collected from pods of each accession every 7 days after anthesis (DPA) and classified in five stages (S1–S5). The S1 pods corresponded to those between 0 and 7 DPA; S2, S3, S4, and S5 to those from 7 to 14, 14 to 21, 21 to 28, and 28 to 72 DPA, respectively. The S5 pods represent the mature fruit stage (Fig. 2B). Pods were frozen in liquid nitrogen immediately after harvest and stored at −80 °C until analysis of either bixin contents or nucleic acids.

### Bixin analysis in achiote seeds

The bixin content was analyzed in seeds throughout their developmental process (Rodríguez-Ávila et al., 2011a), as the total bixin contents and by its cell localization in the tissue sections.

### Chromatographic analysis of bixin

The bixin contents was determined by high-pressure liquid chromatography (HPLC). Extracts were obtained from 10 mg of frozen, dried, and powdered seed tissue from at each developmental stage that were mixed with 0.8 mL of water/methanol 1:1 (v/v). The mixture was shaken for 1 h at room temperature and then mixed 800 μL of chloroform. The tubes were vortexed, centrifuged at 9,615*g* for 10 min at 4 °C (Sorvall Leyend Micro 21R centrifuge; Thermo Scientific, Waltham, MA, USA) and the bottom phase (nonpolar extract) was collected. The extract was then dried and resuspended in 500 μL of chloroform. A 20 μL sample of each extract was injected with nitrogen into a Hypersil ODS C-18 reversed phase column (25 cm × 4.6 mm; five μm diameter beads). The mobile phase consisted of solvent A (0.1% formic acid in water, pH 3) and solvent B (acetonitrile). The column was developed as follows: Step 1, 95% solvent A at injection for 5 min; Step 2, linear increase to 95% solvent B in 25 min; Step 3, 95% solvent A for 4 min; and Step 4, return to 100% solvent B. The samples were detected at 450 nm on an Agilent 1100 MWD detector at a flow rate of 0.8 mL/min at 20 °C. All samples were filtered prior to injection using Millex-GV13 filters (Durapore PVDF, 13 mm diameter, 0.22 μm pore size; Millipore, Billerica, MA, USA) prior to injection. A bixin standard (ChromaDex, Irvine, CA, USA) was used to generate a calibration curve. The samples were quantified based on their column retention time relative to the known bixin standard and by the absorption spectra of individual peaks.

### Cell localization of bixin in achiote seeds

The cell localization of bixin was directly observed as an orange stains in sections from N4P seeds through development. The collected seeds were placed in formalin-acetic acid-alcohol for 24 h, with 15 min periods under vacuum (500 mm Hg) (GE Motor Industrial Systems Mod. 5KH36KNA510X, Wayne, IN, USA), until the seeds sank into the solution (Javelle, Marco & Timmermans, 2011). After that, seeds were dehydrated in an increasing ethanol series (10%, 30%, 50%, 70%, 85%, 95%, 100%; 1 h in each) and then treated with ethanol/histo-clear (3.1, 1:1, 1:3 v/v) for 1 h, followed by histo-clear (100%) (Histo Choice Clearing H2779-1L; Sigma, St. Louis, MO, USA). Several seeds from three different N4P plants (biological replications) were analyzed in triplicate and embedded in paraffin at 60 °C, then placed inside the paraffin block, and stored at 4 °C. The paraffin block was cut with a microtome (Microm International GmbH, Thermo Fisher Scientific, Walldorf; Germany) at a thickness of five μm; the slices were observed using an optical microscope (10× and 100×) (Revelation 111; LW Scientific, Lawrancevilla, GA, USA) and images of the seed morphology were obtained (Javelle, Marco & Timmermans, 2011).

Lycopene extracts from bacteria expressing *BoCCD* candidates ware analyzed with an Agilent 1200 photodiode array detector-UV/VIS, as previously described (Rodríguez-Ávila et al., 2011b). Briefly, 10 μL of the sample was injected into a Hypersil ODS C-18 reversed–phase column (25 cm × 4.6 mm with five-μm diameter beads) and separated at a flow rate of 1 mL/min and 25 °C. Lycopene was detected at 450 and 505 nm. The mobile phase consisted of solvent A (acetonitrile/methanol/isopropanol (75:10:15 by volume)) and solvent B (acetonitrile). The column was developed as follows: Step 1, injection of 100% solvent A for 30 min; Step 2, linear increase to 100% solvent B in 10 min; Step 3, return to 100% solvent A for 3 min prior to the next injection. The lycopene standard was purchased from Sigma-Aldrich (L-9879; St. Louis, MO, USA) and was dissolved in chloroform. All reagents were of HPLC grade.

### Molecular analysis

Four CCD1 members and four CCD4 members (BoCCD1-1 to -4 and BoCCD-4-1 to 4, respectively) were previously isolated as potential bixin biosynthetic genes in a *B. orellana* seed transcriptome (Cárdenas-Conejo et al., 2015). Expression of the eight genes was followed in seeds through development in order to select those coinciding in time and space with bixin accumulation for further characterization.

### Bioinformatic analysis of BoCCD’s

**Figure 3:**
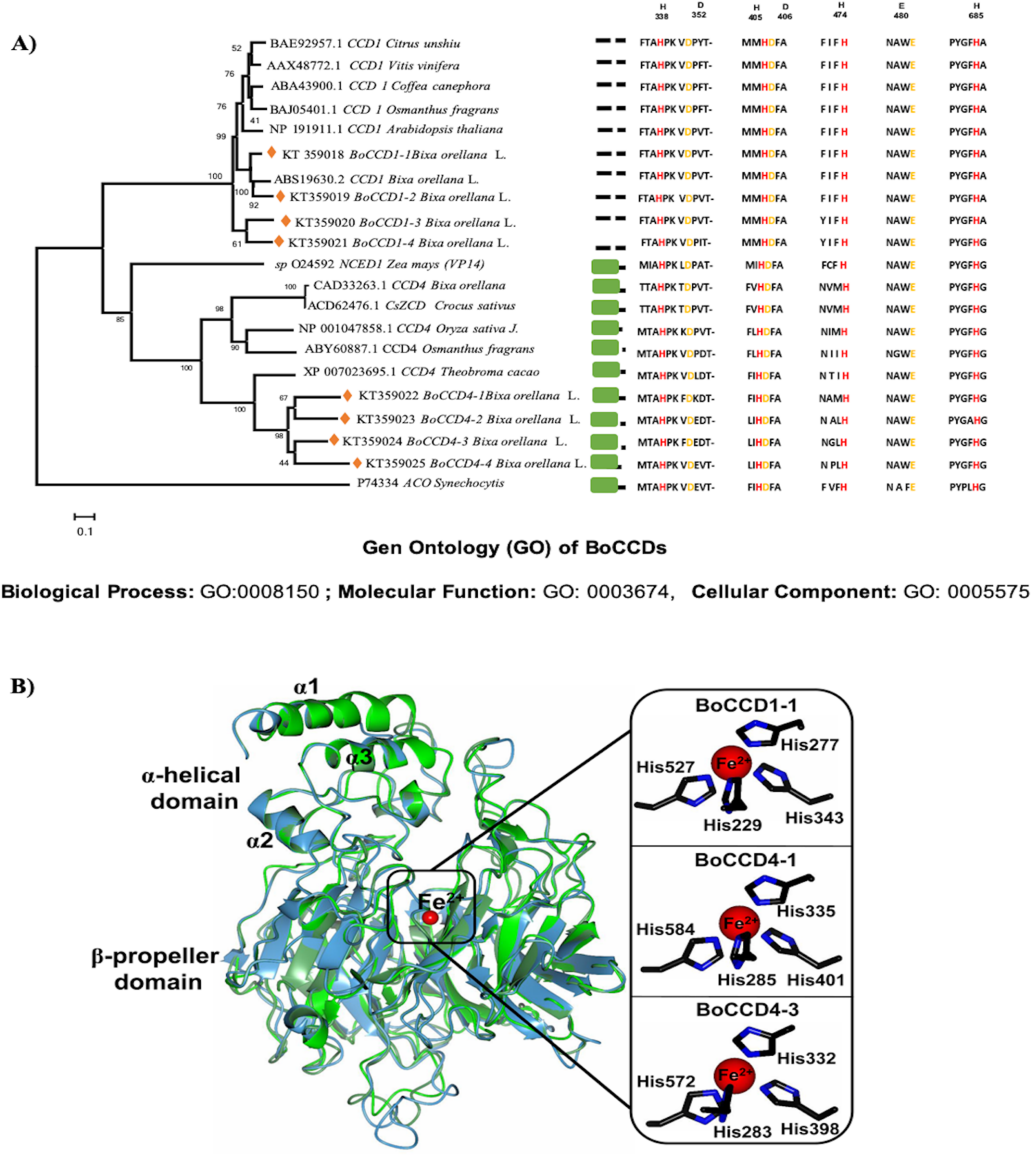
In silico analysis of BoCCDs. (A) Sequence alignments of CCD enzymes, alignment of *B. orellana* BoCCD-like proteins and subcellular locations of BoCCDs by iPSORT. The phylogenetic tree was inferred using the maximum-likelihood method based on the Jones–Taylor–Thornton (JTT) substitution model and was gamma distributed with Invariant sites (G + I) in MEGA6. *Osmanthus fragrans* (ABY60887.1), *Oryza sativa* Japonica group (NP_001047858.1), *Crocus sativus* (ACD62476.1), *Theobroma cacao* (XP_007023695.1), *Z. mays* (O24592), *Citrus unshiu* (BAE92957.1), *Vitis vinifera* (AAX48772.1), *Coffea canephora* (ABA43900.1), *O. fragrans* (BAJ05401.1), *B. orellana* L. (CAD71148.1, ABS19630.2, KT359018, KT359019, KT359020, KT359021, KT359022, KT359023, KT359024), and *Arabidopsis thaliana* (NP_191911.1). Numbers near the branch points represent the bootstrap value produced by 1,000 replications. Phylogenetic trees were rooted with *Synechocystis* apocarotenoid cleavage oxygenase (ACO) (P74334). Orange diamonds in the tree indicate the sequences in the study. The representative alignment indicates the signal peptide in the green rectangle according to iPSORT. Red letters indicate residues of histidine, and yellow letters indicate residues of aspartate or glutamate. Gene ontology (GO) annotation was performed with Blast2GO software for InterPro scanning to determine potential function of BoCCDs. Top 10 GO description in the three main categories, biological process, molecular function, and cellular component (See Dataset3, Götz et al., 2008). (B) Structural superposition of BoCCD1-1 (green), BoCCD4-1 (light blue), and BoCCD4-3 (lawn green) homology models showing the catalytic iron (red sphere) and α-helical and β-propeller domains. Right inset, close-up of four Fe^2+^-coordinating histidine residues (black cylinders).

Eight CCD’s sequences that were previously selected from the *B. orellana* seed transcriptome of accession NP4 because they were more highly expressed in the immature seed (Cárdenas-Conejo et al., 2015), were used to construct a phylogenetic tree, together with CCDs from other organisms (see Fig. 3A for the a list). The maximum-likelihood method, based on the Jones–Taylor–Thornton substitution model (Jones, Taylor & Thornton, 1992), and gamma distribution with invariant sites (G + I) were used. In both cases, the analysis was carried out using algorithms that were included in MEGA6 (Tamura et al., 2013), and the substitution models were predicted by the best-fit substitution model (ML) function included in MEGA6. Phylogeny tests were conducted using the bootstrap method (1,000 replicates). All positions containing gaps and missing data were eliminated. Amino acid sequence alignments were performed with the ClustalW algorithm using MEGA6 default parameters. The phylogenetic trees were rooted with the *Synechocystis* apocarotenoid cleavage oxygenase. Prediction of the BoCCD subcellular localization was performed with the iPSORT server (Bannai et al., 2002). The BoCCD1-1, BoCCD4-1, and BoCCD4-3 enzymes were subjected to BLAST analysis against the Protein Data Bank to search for a suitable template for homology modeling. The crystallographic structure of the VP14 enzyme from *Zea mays* (PBD entry 3NPE) (Messing et al., 2010), was selected as a template because it shares an average sequence identity of ∼38% with the BoCCD1-1, BoCCD4-1, and BoCCD4-3 enzymes was selected as a template.

Homology models were generated using the SWISS-MODEL server (Biasini et al., 2014) and were selected according to the GMQE and QMEAN statistical parameters. The best models for the BoCCD1-1, BoCCD4-1, and BoCCD4-3 enzymes were subjected to energy minimization using YASARA software (Krieger et al., 2009) and validated using MolProbity (Chen et al., 2010). Structural analysis was performed by manual inspection using Coot (Emsley et al., 2010). Graphical representations were generated using CCP4mg version 2.10.6 (McNicholas et al., 2011).

### Gene expression by quantitative reverse transcription-PCR

Total RNA was isolated from the five developmental stages in triplicate from three different N4P and P13W individuals. Seeds (50 mg) were ground in liquid nitrogen using a mortar and pestle. The PureLink^®^ RNA Mini Kit was used (Cat. No. 12183018A; Ambion^®^, Carlsbad, CA, USA), and the resulting RNA was subsequently treated with DNase (DNase I amplification grade, Cat. No 18068-015; Invitrogen, Carlsbad, CA, USA). The extracted RNA was stored at −80 °C until analysis (Rodríguez-Ávila et al., 2009). The RNA quality was verified in 1.2% agarose gels that were stained with ethidium bromide. Reverse transcription was performed with SuperScript™ III reverse transcriptase (Cat. No. 18080-093; Invitrogen, Carlsbad, CA, USA) using 100 ng of total RNA from each tissue.

Specific primers were designed for each one of BoCCD members, considering differences in the amplified targeted region of each specific member (Table 1). Expression analysis was conducted by reverse transcription-PCR (RT-qPCR). The reaction mixture contained 100 ng of cDNA and the SYBR^®^ Green qPCR SuperMix-UDG (Cat. No. 11733046; Invitrogen, Carlsbad, CA, USA). PCR was performed using an iCycler IQ real-time PCR detection system (Bio-Rad, Hercules, CA, USA). The amplification program included 35 cycles (30 s each) at 95 °C for DNA denaturation, followed by 62.3 or 57.6 for primer annealing, and 72 °C for extension. The PCR program included an initial 2 and 4 min periods at 50 and 95 °C, respectively, to activate the polymerase, and a final 10 min extension at 72 °C. Alternative primer alignment temperatures corresponded to either *BoCCD1s* or *BoCCD4s* target sequences (Table 1). The expression of the 18S rRNA gene was followed in each sample as an internal reference, taking leaves to standardize comparisons among gene expression of seeds development stages. The specificity of the PCR was assessed by the presence of a single peak in the dissociation curve performed after the amplification. Each quantitative PCR experiment was run three times separately and included three replicates to calculate the standard error for each sample. The results were analyzed by the 2^−ΔΔCT^ method (Livak & Schmittgen, 2001) with appropriate validation experiments (Bio-Rad Laboratories, 2006). All data were analyzed using two-way ANOVA, followed by a multiple-comparisons *T*-test (*P* = 0.05), and multifactorial analysis was performed to correlate the level of expression with bixin accumulation, followed by the Person correlation test. The correlation index varied in the range of (−1 to 1), with values closer to 1 indicating a positive correlation.

**Table 1:** Oligonucleotides.

Oligonucleotides	5′ sequence 3′	*T*_m_	Size (bp)	GenBank accession
F-BoCCD1-1	CTGGCACTTAACGAGGGT	62.3	127	KT359018
R-BoCCD1-1	CAACCTTAGGATGAGCAGTG			
F-BoCCD1-2	CTGGCACTTCAAGAGGCA	62.3	127	KT359019
R-BoCCD1-2	CAACCTTAGGATGAGCAGTG			
F-BoCCD1-3	TCCCAACCCAAAGTTTCAC	57.6	198	KT359020
R-BoCCD1-3	TCCTATGCTTACCATGAGTGG			
F-BoCCD1-4	TGCCAATATGGACGAGTCC	57.6	214	KT359021
R-BoCCD1-4	TAGCCATCATCCTCCTCCA			
F-BoCCD4-1	AGCTTCCACCGTCTCTCCA	57.6	200	KT359022
R-BoCCD4-1	AATGATCGCAGCTCCTCTGC			
F-BoCCD4-2	GATTCCCACCTCTCTGGA	57.6	213	KT359023
R-BoCCD4-2	AACATATTGGGCATGCGA			
F-BoCCD4-3	ATGAGGACACCAAGGACG	57.6	213	KT359024
R-BoCCD4-3	CTAGCATCATTTTGGCAACG			
F-BoCCD4-4	TACTGCCAAGATGATCTGG	57.6	153	KT359025
R-BoCCD4-4	GCATTGAGGACATGTAATGG			
F-18S	CGGCTACCACATCCAAGGAA		200	AF206868
R-18S	GCTGGAATTACCGCGGCT3			
ORFs				
BoCCD1-1F	TTACTCGAGATGGCTCAGGAGGCGGAGAAGC	61	1,656	KT359018
BoCCD1-1R	ATACTCGAGGTCTTGCCTCAAGATCTCTCCATGTG			
BoCCD4-1F	CATCATATGTTCTTCCTTCGCATGATG	53	1,846	KT359022
BoCCD4-1R	AAAGGATCCGTGACTAACATAGGAAATCTCC			
BoCCD4-3F	CATCATATGTCCTCACAGAAGATGTACTG	58	1,785	KT359024
BoCCD4-3R	AAAGGATCCTAGTCAAGGAGTATGCACACGA			

### In situ histological localization of BoCCDs RNA

In situ RT-PCR was performed to identify the specific cells expressing the selected BoCCD candidates (*BoCCD1-1*, *BoCCD4-1*, and *BoCCD4-3*). Thick slices of the seeds sections (50 μm) were obtained as described above and washed twice histo-clear for 10 min, deparaffinized, and hydrated in a series of alcohol concentrations (100%, 95%, 85%, 70%, 50%, 30%, and 10%) for 30 s and were finally washed with DEPC water. This procedure was followed by washing with 1× PBS (phosphate-buffered saline) for 2 min and treatment with protease (one mg/mL) for 20 min at 37 °C. The enzyme was then neutralized with 1× PBS + glycine (0.2%) for 2 min and the samples was washed again with 1× PBS for 2 min (Javelle, Marco & Timmermans, 2011). Subsequently, the tissue was treated with DNase (DNase I amplification grade, Cat. No. 18068-015; Invitrogen, Carlsbad, CA, USA). Next, in situ RT-PCR was performed using the whole tissue with a SuperScript™ III One-Step RT-PCR system (Cat. No. 12574026; Invitrogen, Carlsbad, CA, USA) in a 25 μL reaction volume consisting of 2× reaction mix buffer, DIG-dUTP DIG-11-dUTP, alkali-labile, tetralithium salt, 1 mMDIG-11-dUTP, alkali-labile, tetralithium salt, 1 mM (Cat. No. 11573179910, DIG-11-dUTP, alkali-labile, tetralithium salt, 1 mMDIG-11-dUTP, alkali-labile, tetralithium salt, 1 mM Roche, Mannheim, Germany), anti-Dig-AP (Cat. No. 11093 274910, Roche, Mannheim, Germany), and DIG Wash and Block Buffer Set (Cat. No. 1158576200, Roche, Mannheim, Germany). Immediately after reaction, the tissues were treated with the DIG Wash and Block Buffer Set (Cat. No. 11585762001; Roche) and observed with a Zeiss Axioplan microscope (10×, 20×, 100×).

### In vitro functional analysis of BoCCDs

BoCCDs with expression patterns that matched the bixin time and spatial distributions (see Results) were selected for further characterization via heterologous expressed in *E. coli* cells because they are potential participants in bixin biosynthesis.

### Heterologous expression and in vitro functional analysis of BoCCDs

BoCCD1-1, BoCCD4-1, and BoCCD4-3 were assessed for lycopene cleavage activity through their expression in a lycopene-accumulating bacterial strain. For this, the BL21 *E. coli* strain bearing the plasmid pACCRTEIB was employed. This plasmid confers the capacity of lycopene biosynthesis and would produce an orange-reddish color in the bacterial cells. The color should fade away if the lycopene is consumed as a substrate of any of the heterologously expressed BoCCD’s. The complete ORFs were amplified using specific primers (Table 1) and were introduced into the cloning vector pCR™8/GW/TOPO (Cat. No. K2500-20; Invitrogen, Carlsbad, CA, USA). Each of the cloned ORFs was then individually introduced into the expression vector pDEST™17 under the control of an arabinose-inducible promotor (Gateway Recombination Cloning Technology; Cat. No. 11803-012; Invitrogen, Carlsbad, CA, USA). Double recombinants (pDEST17BoCCD1-1, pDEST17BoCCD4-1, and pDEST17BoCCD4-3) were selected on ampicillin and chloramphenicol containing media (100 and 50 μg/mL, respectively), and positive colonies were grown in five mL a Luria–Bertani (LB) liquid medium overnight at 37 °C. Whole cell were used to inoculate 100 mL of LB medium, supplemented with the same antibiotic composition. Cultures were incubated overnight at 24 °C, before inducing expression by adding arabinose to a final concentration of 0.2% and incubating for 16 h with gentle shaking, following the manufacturer’s instructions (Cat. No. 11801-016; Invitrogen, Carlsbad, CA, USA). After induction, the cells were collected by centrifugation at 135*g* (Universal 32 R; Hettich zentrifugen, Tuttlingen, Germany) for 10 min in 50 mL Falcon tubes, and the color of the bacterial pellet was visually assessed, both for the recombinants and for the lycopene-accumulating strain (pACCRTEIB). Fading of the expected orange tone indicated lycopene cleavage (see Results) (Cunningham et al., 1996; Misawa et al., 1990; Simkin et al., 2004).

### LC-MS/MS analysis

The correct formation of the lycopene oxidative cleavage product (bixin aldehyde) by the recombinant BoCCD’s was analyzed for in the bacterial extracts by UHPLC-ESI-QTOF-MS/MS. Bacterial pellets were resuspended in five mL of chloroform/ethanol (2:1) and vortexed for 10 min, pellet was completely dissolved. After centrifugation, the supernatant was collected, dried under a nitrogen atmosphere and stored at −80 °C until analysis. Fragmentation patterns in extracts from bacteria displaying positive lycopene cleavage activity were analyzed in a Waters XEVO-G2XSQTOF quadrupole time-of-flight mass spectrometer (Milford, MA, USA). The extracts were directly injected into the quadrupole MS at a flowrate of 5 μL/min, setting the mass detection range at 100–1,000 *m/z*, with an average reading time of 1 min and an ESI-positive Source. The MS parameters were set as follows: ESI source in positive ion mode; capillary voltage: 32 kV; sampling cone: 42; source offset 80; and N_2_ cone gas flows: cone gas 50 1/h: desolvation gas 5000 MS/MS cone voltage: 35V and energy collision: 25V dry gas.

Alternatively, extracts were previously separated by UHPLC on a C18 ACQUITY UPLCrBEH column (1.7 μm) maintained at 25 °C, with a 0.4 μL/min flow of the mixtures acetonitrile:Ispropanol:methanol (75:10:15, v/v/v; **A**) and 99% formic acid in acetonitrile (v/v; **B**) run as a gradient (A:B) 25:75 to 15:85 over the first 3 min; then at 10:90 up to 5 min; and at 25:75 up to 20 min. The column eluate was split to allow only 0.45 mL/min to enter the ESI interface. The mass spectra were acquired with a scan range from 100 to 1,000 *m/z*; using the same setting as above.

### Identification of the functional groups in BoCCD cleavage products

Functional groups in the oxidative cleavage products of the recombinant bacterial expressing the different BoCCDs were determined by Fourier transform infrared (FTIR) absorption Spectra (range from 4,000 to 500 cm^−1^) using a Cary 630 FTIR portable spectrometer equipped with a five-bounce zinc selenite ATR accessory (Agilent Technologies Inc., Santa Clara, CA, USA). The bacterial extracts were pDEST17BoCCD1-1 and pDEST17BoCCD4-3 and standard bixin was used as a control.

## Results

### Sequence analysis and homology modeling of BoCCDs

The complete amino acidic sequences of the eight *B. orellana* CCDs, that were previously identified in the seed transcriptome were used in an unrooted phylogenetic tree (Fig. 3A). The CCDs were divided into two well-defined groups: BoCCD4s and BoCCD1s (Fig. 3A). The sequence alignments revealed the presence of four histidine residues that are involved in coordinating the Fe^2+^ in 2-oxoglutarate-dependent dioxygenases, among other conserved motifs (Kloer & Schulz, 2006; Priya et al., 2016) (Fig. 3B). As expected for CCD1s, which are cytosolic enzymes, no transit peptide at the N-terminus was observed in any according to iPSORT prediction (Wei et al., 2016). This was in contrast with the BoCCD4s, in which a 30- residues transit peptide was predicted at the N-terminus. This putative transit peptide presented the characteristic domain present in lignostilbene-alpha, beta-dioxygenases, and related enzymes (Marasco & Schmidt-Dannert, 2008; Rubio-Moraga et al., 2014; Rubio et al., 2008), typically found in chloroplast targeted proteins (Fig. 3A). However, no membrane anchoring peptides were predicted in the protein sequence, suggesting that BoCCD4s could cross the chloroplastic membrane without being retained in them (Wei et al., 2016). Notably, lycopene oxidation to bixin is proposed to take place inside plastids bixin storage cells (BSC) (Louro & Santiago, 2016).

### Bixin accumulation follows a developmental-associated pattern in *B. orellana* seed arils

**Figure 4:**
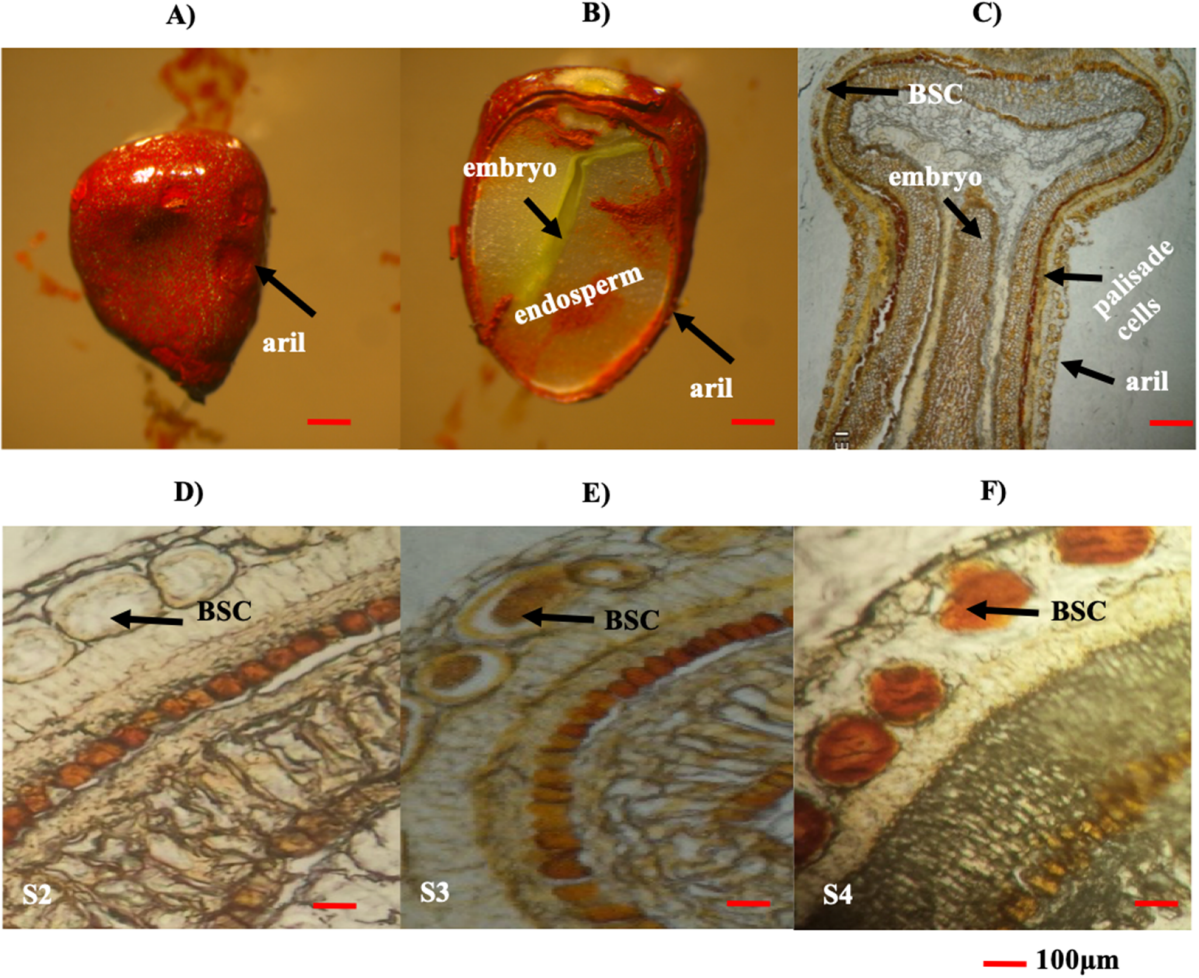
Histological analysis of *B. orellana* L. seeds. (A) Immature seed, (B) immature seed longitudinal cut, (C) immature seed longitudinal cut in paraffin, (D) S2 structure, (E) S3 structure, (F) S4 structure. BSC, bixin storage cell. Bar 100 μm. Photos by Victor Manuel Carballo-Uicab.

The seed aril is a membranous tissue that covers the seed in some species. In *B. orellana* this tissue is the site of bixin synthesis and accumulation in the BSCs. Throughout development, the bixin contents steadily increased in the seeds of two different accessions, up to the premature stages (S1–S4; Table 2). However, in mature seeds (S5), the bixin content decreased to about a 40% of the maximum (Table 2). Variations in the number and size of the seeds were also recorded (Table 2). Although the bixin contents followed similar trends throughout seed development in both accession, values were consistently higher (between 20% and 57%) in NP4 than in P13W (Table 2). Microscopic examination showed that BCSs formed circular clusters in the outer aril layer, coinciding with the increase in bixin throughout the first four developmental phases (Figs. 4A–4F). Once the mature stage was reached, the pigment was excreted from the seed aril, since it could also be observed in different seed areas. This could account for the decreased contents noticed in the mature seeds (Figs. 4D–4F).

**Table 2:** Characterization of seeds and bixin content.

Accession	Seed stage	DPA	Length (mm) (SD)	Width (mm) (SD)	Weight (mg) (SD)	Bixin (mg/g DW) (SD)
N4P	S1	0–7	2 (0)	1.6 (0.41)	7.4 (0.0003)	7.1 (0.2)^c^
	S2	7–14	2.9 (0.22)	1.5 (0.35)	8.6 (0.0002)	9.2 (0.2)^b^
	S3	14–21	3.7 (0.44)	2.2 (0.27)	11.3 (0.0002)	11.5 (0.2)^a^
	S4	21–28	4.1 (0.22)	2.3 (0.44)	26.1 (0.0005)	12.9 (0.9)^a^
	S5	28–42	4.5 (0.21)	4 (0.40)	32 (0.0017)	6 (0.3)^c^
P13W	S1	0–7	1.8 (0.27)	1 (0)	6.9 (0.001)	4.5 (0.2)^c^
	S2	7–14	3 (0)	1 (0)	7.6 (0.0006)	5.5 (0.2)^bc^
	S3	14–21	3.9 (0.22)	2 (0)	1.2 (0.0009)	6.6 (0.2)^b^
	S4	21–28	6 (0)	3.5 (0.3)	23.1 (0.0009)	10.1 (0.8)^a^
	S5	28–42	4.8 (0.2)	3.4 (0.32)	26 (0.0017)	4.1 (0.3)^c^

**Notes:**

Values are presented as the mean (SD) of three replicates for bixin and five replicates for length, width, and weight. Days after anthesis (DAP).

Comparison of two accessions *B. orellana* (P13W and N4P) based on data: developmental stage of *B. orellana* seeds of 7 days difference growth, size (length and width), weight and bixin content (mg/g DW). In bixin quntification, similar letters were not significantly different (*P* = 0.05) by two-way ANOVA followed by a multiple-comparisons *T*-test.

### Expression of *BoCCD1-1, 4-1*, and *4-3* are related to the developmental accumulation of bixin *B. orellana* seeds

**Figure 5:**
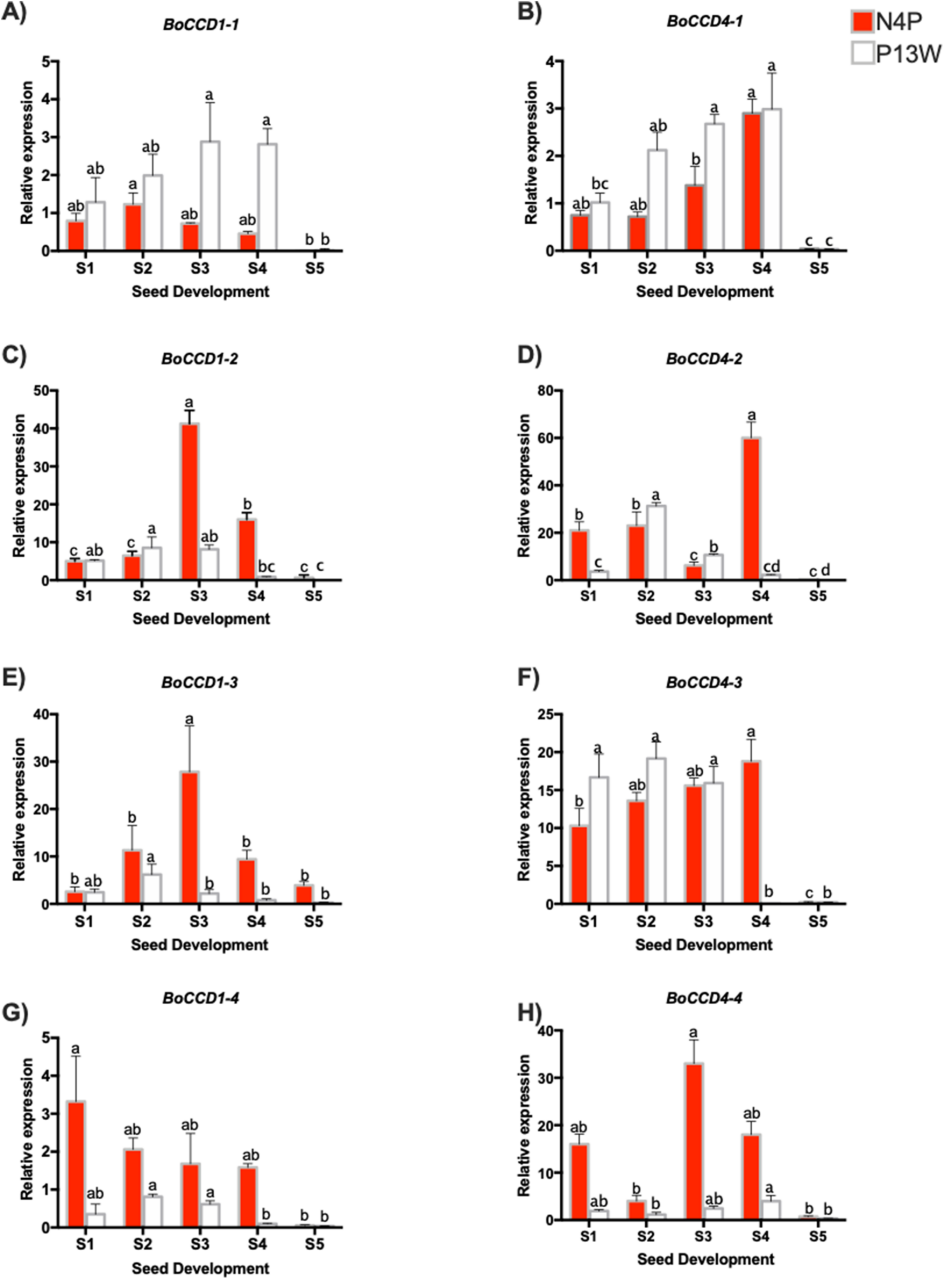
Transcript levels of BoCCD genes throughout *B. orellana* L. seed development. (A–H) Transcript levels of BoCCDs in the N4P (red bar) and the P13W (white bar) accession. The expression values are relative to leaf (external control), which was set to 1 and the constitutive 18S gene. The *X*-axis represent the seed stages: S1, S2, S3, S4, and S5. Error bars are standard errors of the mean from three technical replicates, *P* = 0.05 level. Bars with similar letters were not significantly different (*P* = 0.05) by two-way ANOVA followed by a multiple-comparisons *T*-test.

Eight *B. orellana* CCDs that were potentially involved in bixin biosynthesis were identified in a seed transcriptome obtained from a local cultivar based on their similitude to lycopene cleavage dioxygenase (LCD) from different species, including *B. orellana, Arabidopsis thaliana, Theobroma cacao* (Cárdenas-Conejo et al., 2015). Four CCDs each belonged to the CCD4 and CCD1 families and were named *BoCCD4-1* to -*4* and *BoCCD1-1* to -*4*. The expression profiles through seed development of these eight candidates were followed by RT-qPCR in both the N4P and P13W accessions (Figs. 5A–5H).

Among the *BoCCD1* members, *BoCCD1-1* expression displayed a similar pattern to that of bixin accumulation increasing during the first four developmental stages and then drastically decreasing in S5 (Fig. 5A), but this occurred only in P13W accession. In fact, a strong correlation was found in P13W (Pearson’s *r* = 0.65) in comparison to N4P (Pearson’s *r* = 0.19). Expression profiles of the remaining *CCD1* candidates (-*2*, -*3*, and -*4*) did not match those of bixin accumulation in either P13W nor N4P, showing consequently poor correlations (Pearson’s *r* between −0.6 and −0.185) (Figs. 5A–5H). Interestingly, *BoCCD1-2* and -*3* in N4P S3 seeds displayed the highest expression levels (approximately 10-fold higher than those of *BoCCD1-1*), although with low correlation values (Pearson’s *r* = 0.6 and 0.5, respectively) (Figs. 5C and 5E). Therefore, we decided to continue to further characterize *BoCCD1-1* for its possible involvement in bixin biosynthesis.

On the other hand, to identify the *BoCCD4* genes potentially involved in bixin synthesis, the expression levels of *BoCCD4-1* to -*4* were also profiled throughout seed development. *BoCCD4-1* expression increased during seed development in both accessions, following a trend similar to that of bixin accumulation and showing a highly significant correlation (Pearson’s *r* = 0.87 and 0.7 for N4P and P13W, respectively; Fig. 5B). No other *BoCCD4* gene showed an expression pattern that was similar to bixin accumulation in either P13W or N4P, except for *BoCCD4-3* which displayed high expression levels beginning in stage S1, with a high correlation (Pearson’s *r* = 0.89) in N4P accession (Fig. 5F).

All *BoCCD1s* and *BoCCD4s* genes expression levels declined in the S5 stage, corresponding to decreased bixin accumulation in seeds at this stage. Overall, the expression levels of *BoCCDs* were higher in seed stages S3 and S4.

Overall, the expression profiles of only three, out of the eight genes initially considered as candidates, displayed significant correlation with bixin accumulation throughout seed development. These were *BoCCD1-1*, *BoCCD4-1*, and *BoCCD4-3* and were selected for further characterization as bixin biosynthetic genes.

### Expression of *BoCCD4-3* and *BoCCD1-1* expression occurs in the bixin storage cell, concomitantly with bixin accumulation

**Figure 6:**
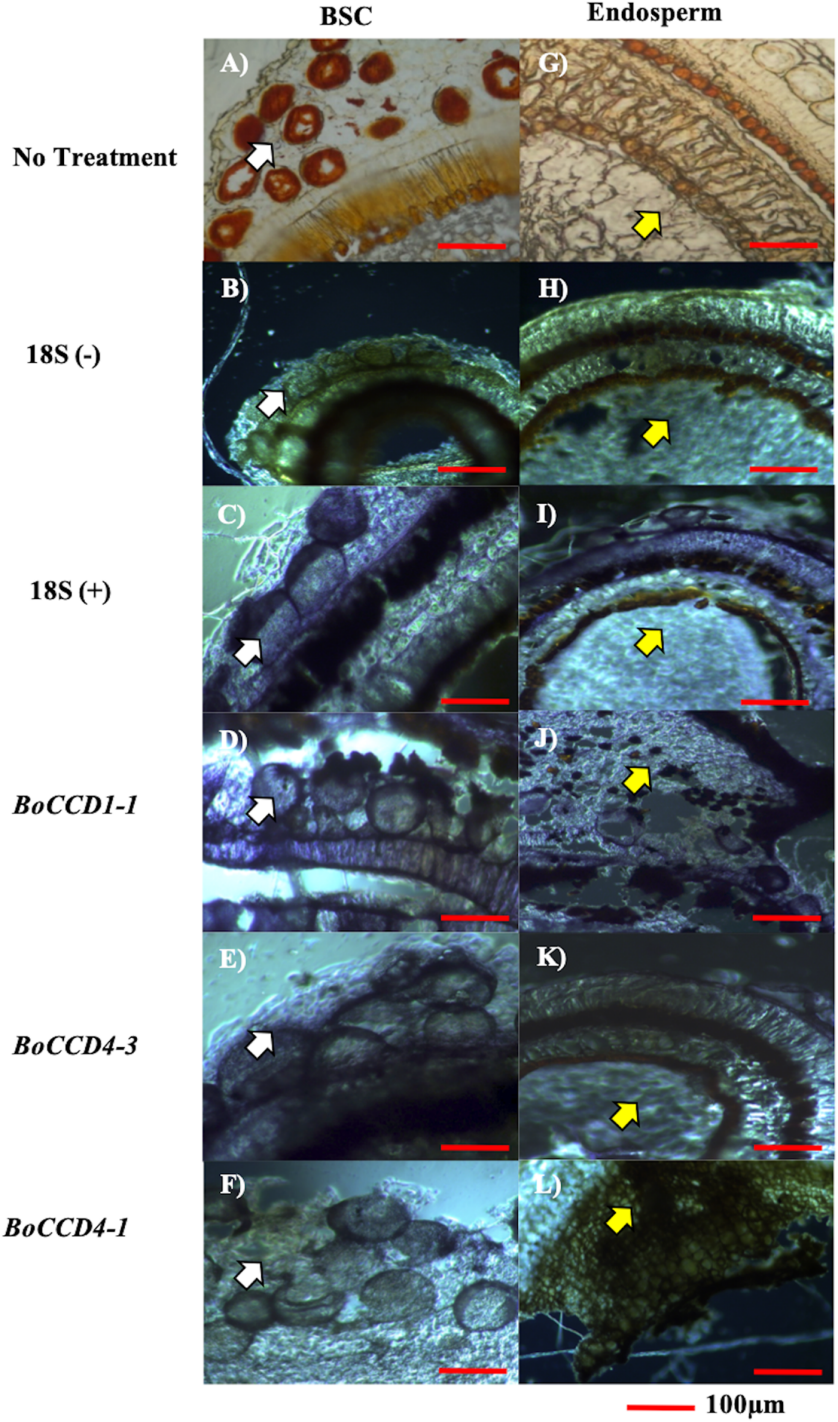
In situ RT-PCR of selected BoCCDs. The blue strain indicates gene expression. (A and G) Immature seed without treatment, (B and H) 18S negative control, (C and I) 18S positive control, (D and J) BoCCD1-1 gene, (E and K) BoCCD4-3 gene, and (F and L) BoCCD4-1 gene. white arrow: BSC (bixin storage cells), yellow arrow: endosperm, Bar 100 μm. Photos by Victor Manuel Carballo-Uicab.

Cell types expressing the three candidate genes (*BoCCD1-1*, *BoCCD4-1*, and *BoCCD4-3*) were located by in situ PCR in S4 seed sections of N4P (Figs. 6A–6L). As expected, transcripts of the three candidates were located in seed sections; however, important differences were noticed. Both *BoCCD1-1* and *BoCCD4-1* were located in BSCs (Figs. 6F–6D white arrow head) as well in the endosperm (Figs. 6J–6L yellow arrow head). However, *BoCCD1-1* was more concentrated in BSCs than in the endosperm. This was different from *BoCCD4-1*, which was comparable in both regions. Interestingly, *BoCCD4-3* expression was markedly higher in BSCs than in the endosperm (Fig. 6E). Transcripts from a constitutively expressed gene (ribosomal 18S), were located in all of the analyzed tissues at similar levels, as expected (Figs. 6C and 6I). In this way, the expression of *BoCCD4-3* and *1-1*, but not that of *4-1*, coincided with bixin accumulation in the BSCs of aril cells and were selected for heterologous expression (Rodríguez-Ávila et al., 2011b).

### *BoCCD1-1* and *BoCCD4-3* catalyzed the oxidative cleavage of lycopene to bixin aldehyde in transformed *E. coli* cells

**Figure 7:**
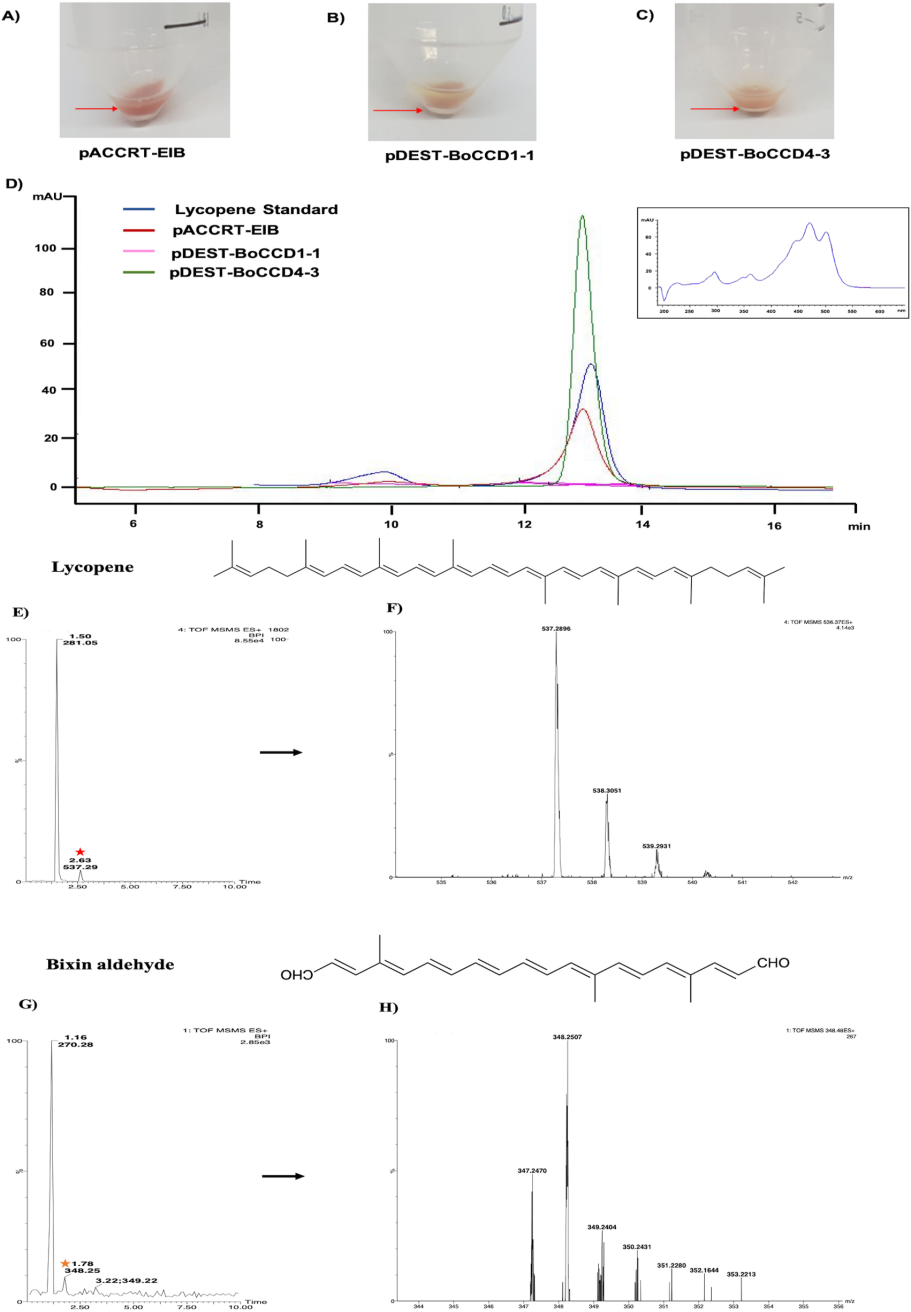
Analysis by HPLC, UHPLC, and MS. Recombinant BoCCDs in *E. coli* total extracts: (A) pACCRT-EIB, (B) pDEST-BoCCD1-1, (C) pDEST-BoCCD4-3, (D) HPLC chromatograms: lycopene standard (blue line), pACCRT-EIB extract (red line), pDEST-BoCCD1-1 (pink line), pDEST-BoCCD4-3 (green line), box: lycopene UV spectrum, UHPLC-MS of pDEST-BoCCD4-3, (E) lycopene chromatogram (red star), (F) lycopene MS spectrum with lycopene detection (*m*/*z* 536.3), (G) bixin aldehyde chromatogram (yellow star), and (H) bixin aldehyde MS spectrum (*m*/*z* 349.2), and *L* = 505 nm, *T* = 25 °C, running time = 30 min.

**Figure 8:**
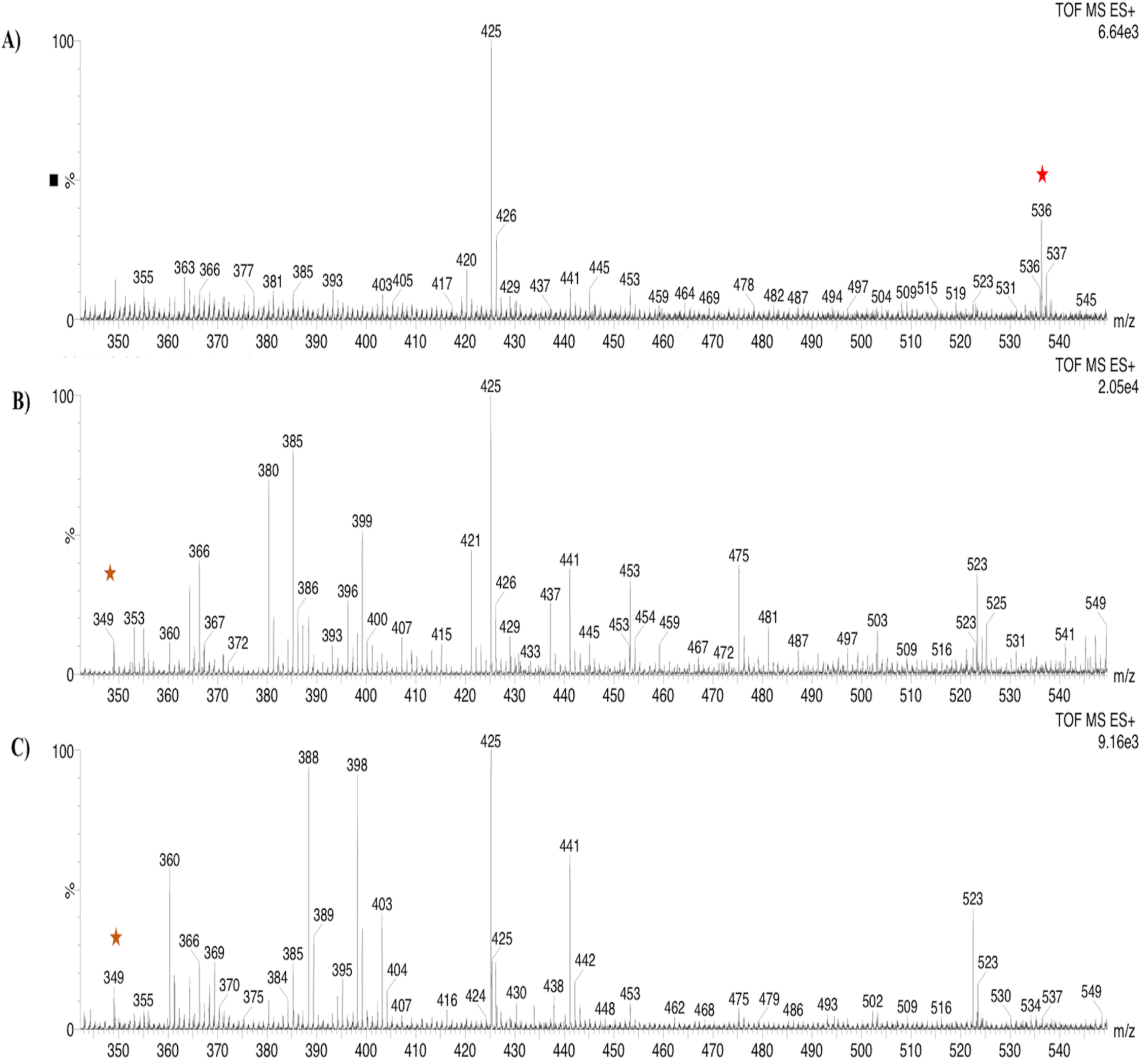
Analysis by UHPLC-ESI-QTOF-MS/MS. Lycopene (*m*/*z* 536.3) (red star) and bixin aldehyde detection (*m*/*z* 349.2) (orange star) in (A) pACCRT-EIB, (B) pDESTBoCCD1-1, and (C) pDEST-BoCCD4-3.

**Figure 9:**
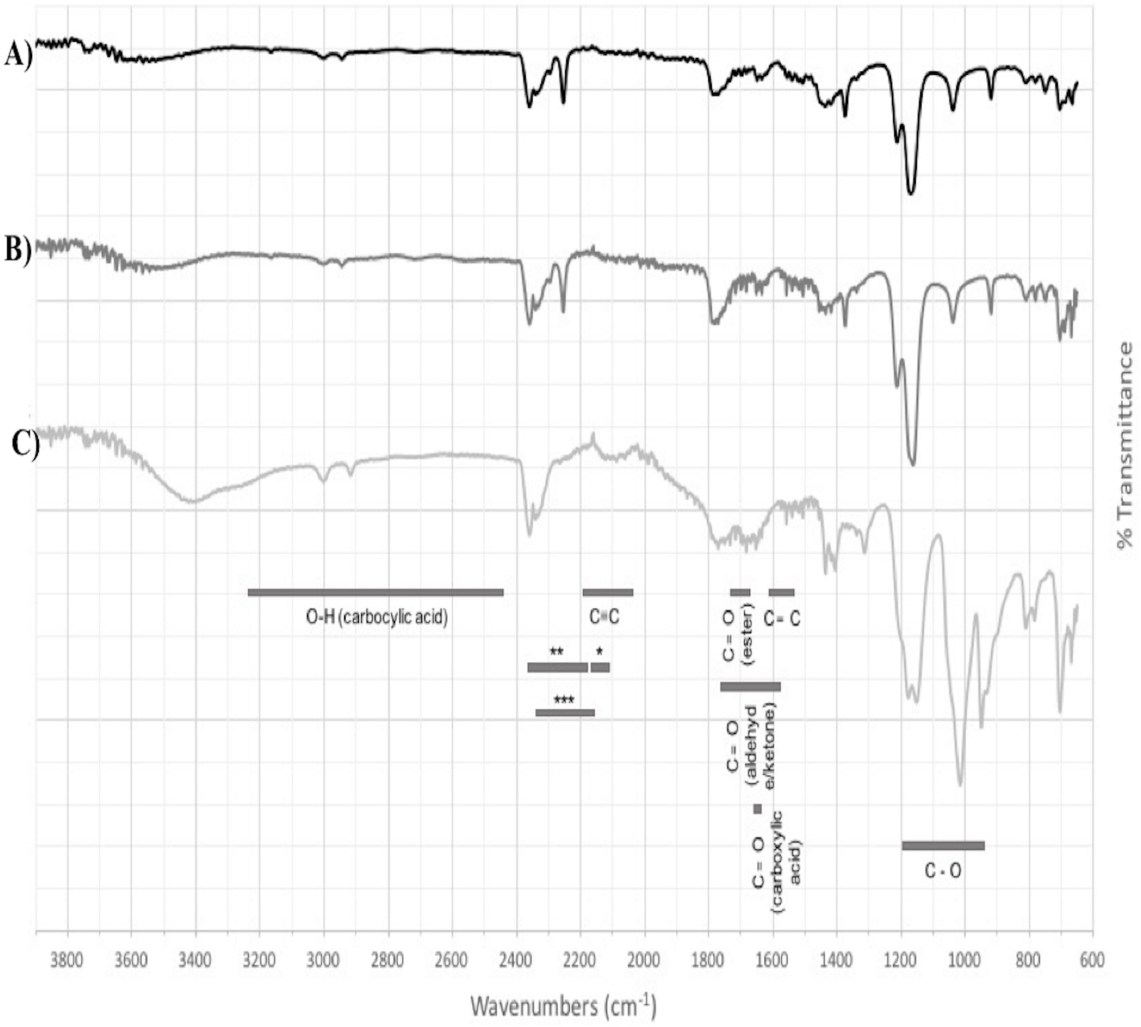
FTIR spectrum. (A) pDESTBoCCD1-1, (B) pDESTBoCCD4-3, and (C) bixin standard as a control of harmonic absorptions of NIR (near-infrared overtone absorption): between CHO and CH (*, **) and between CH_2_ and CH_3_.

Based on the expression profiles and cellular locations, two of the three candidates were selected for functional characterization: *BoCCD1-1* and *BoCCD4-3*. To confirm their involvement, they were heterologously expressed in the pACCRTEIB *E. coli* strain, which was engineered to accumulate lycopene and therefore displays an orange coloration in isolated colonies or cell pellets spun down from suspension cultures (Figs. 7A–7C). The complete ORFs for both *BoCCD1-1* and *BoCCD4-3* were successfully cloned in the expression vector pDEST17 and transformed into the bacterial cells to establish the lycopene cleavage activity. Bacterial cells transformed with the empty vector maintained their coloration after overnight culturing at 37 °C, whereas those bearing the *BoCCD1-1* and *BoCCD4-3* plasmids decolored to a yellowish tone, suggesting lycopene breakdown by the action of the expressed corresponding proteins (Figs. 7A–7C). Moreover, chromatographic analysis of the bacterial extracts revealed that lycopene (Rt 13 min) was not present in extracts from pDESTBoCCD1-1 cells but was present in the empty vector (Figs. 7D–7G). Interestingly, no other compounds were detected in the pDESTBoCCD1-1 extracts, in contrast to those from pDESTBoCCD4-3 cells, where a prominent, slightly slower signal than lycopene was present (Rt 13.3; Fig. 7G). To confirm the identity of lycopene and the alleged derived products, the extracts were analyzed by UHPLC-MS (Figs. 7H–7K). Two major compounds, at *m/z* 536.3 and 349.2, respectively, corresponding to lycopene and bixin aldehyde (Bouvier, Dogbo & Camara, 2003), were detected in pDESTBoCCD4-3 extracts (Figs. 7H–7K). However, these compounds were not observed in the pDESTBoCCD1-1 extracts. This result could be due to either to low compound abundance or an efficient consumption (Rubio et al., 2008). Therefore, the bacterial extracts were directly analyzed by MS/MS in order to increase the sensitivity. Lycopene (*m/z* 536.3) only was identified only in extracts from pACCRTEIB bacteria (transformed with the empty vector) (Fig. 8A), whereas those from pDESTBoCCD1-1 and pDESTBoCCD4-3 cells also presented a signal at *m/z* 349.2, assigned to bixin aldehyde (Bouvier, Dogbo & Camara, 2003) (Figs. 8B and 8C). These results suggest that the selected *BoCCDs* have the capacity to produce bixin aldehyde from lycopene in vitro by oxidative cleavage at positions 5,6 (5′/6′). Formation of the correct cleavage products (bearing aldehyde functional groups) was analyzed by FTIR spectroscopy. Extracts from both pDESTBoCCD1-1 and pDESTBoCCD4-3 were analyzed in the spectral range 4,000 to 500 cm^−1^. A signal at 2,250 cm^−1^ was noticed in the lycopene-incubated extracts from both pDESTBoCCD1-1 and pDESTBoCCD4-3 but not in the bixin standard lacking aldehyde groups (Figs. 9A–9C). Such signal is ascribed to the product of the harmonious absorption between the change of bonds of groups CHO (*), CH (**), and CH3 (***) groups, indicating the different compounds generated by the reaction of lycopene to form bixin aldehyde. Signals corresponding to the triple C bonds were also recorded nearby (2,100–2,250 cm^−1^). There were noticeable differences compared with the bixin control in the spectral ranges corresponding to C–O double bonds, esters, carboxylic acids, and aldehyde and ketone functional groups (1,600–1,800; 1,735–1,800; 1,700–1,725, and 1,630–1,820 cm^−1^, respectively; Figs. 9A–9C). The asymmetry of the two peaks in this spectral region, found in pDESTBoCCD1-1 and pDESTBoCCD4-3, but not in bixin, could indicate the C=O union between the aldehyde (bixin aldehyde) and acids (norbixin) or ester (bixin) groups detected as different masses.

**Figure 10:**
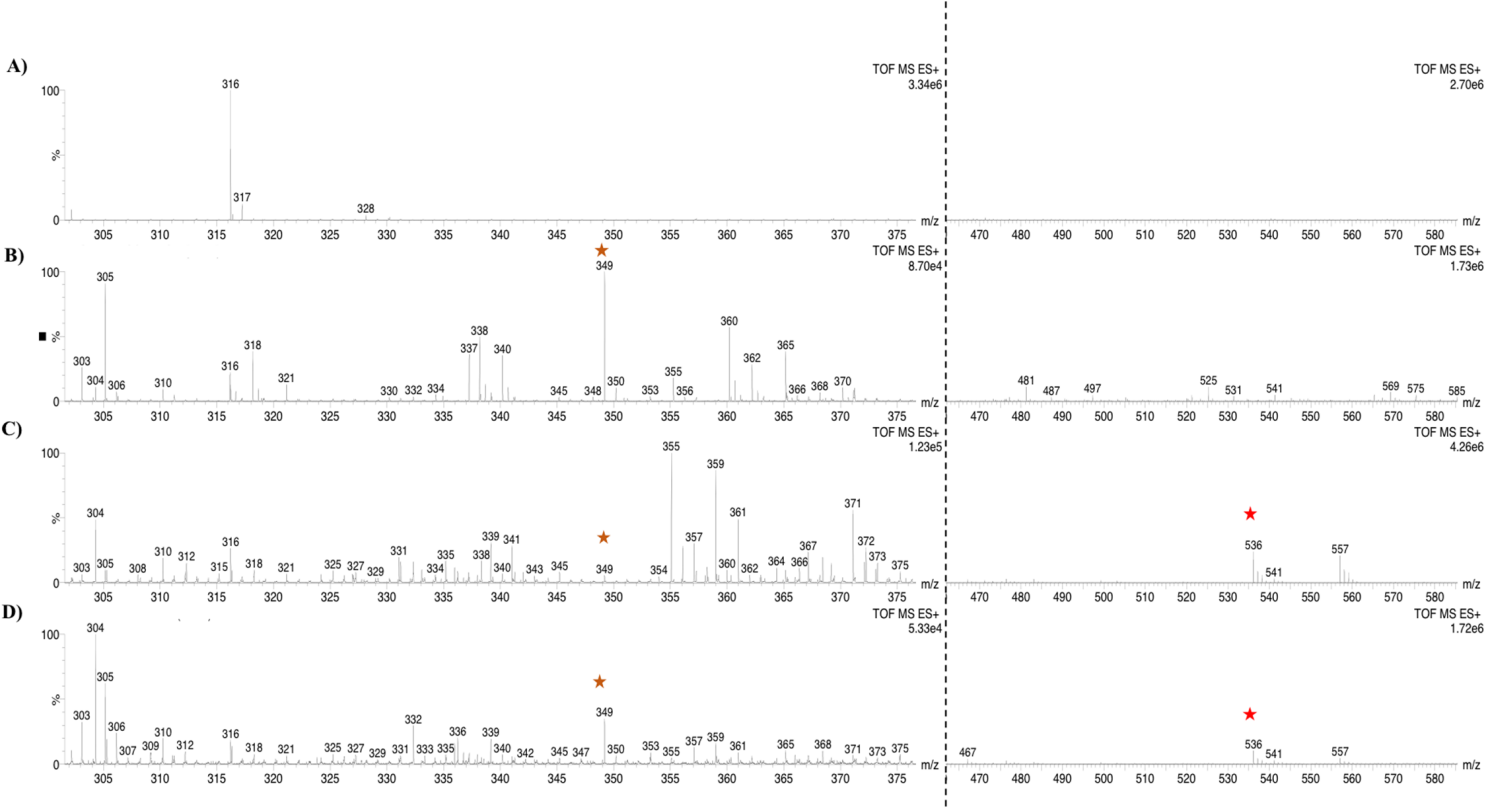
Analysis by UHPLC-ESI-QTOF-MS/MS. Bixin aldehyde detection (*m/z* 349.2) (orange star) adduct [M+H]^+^ and lycopene (*m/z* 536.18) (red star) to 1 min (A) pDESTBoCCD1-1, (B) pDESTBoCCD1-1+LYC, (C) pDESTBoCCD4-3, and (D) pDESTBoCCD4-3+LYC. LYC, lycopene.

To further confirm the lycopene cleavage activity of BoCCD1-1 and BoCCD4-3, extracts from the corresponding recombinant *E. coli* cultures were incubated 1:1 with one mM lycopene for 1 min at 22 °C (Fig. 10). The corresponding bixin aldehyde signal at *m/z* 349, was observed in extracts from pDESTBoCCD1-1 (Figs. 10A and 10B). This was in contrast to that of lycopene (*m/z* 536), which was not detected. This observation suggests the efficient conversion of lycopene into the aldehyde. The lack of lycopene detection in extracts even after 3 min of incubation seems to support this interpretation (DOI 10.6084/m9.figshare.7967204). Interestingly, despite the positive results in the decoloring assay of pDESTBCCD4-3 cultures (Fig. 7C), the bixin aldehyde signal at *m/z* 349.2 was almost imperceptible in extracts obtained from these cultures (Figs. 7H–7K). Moreover, cell-free extracts failed to transform the externally added lycopene into the aldehyde, unless they were incubated for long periods (up to 5 min) and this coincided with a decreasing the lycopene signal (*m/z* 536; DOI 10.6084/m9.figshare.7967204). These results suggest that *BoCCD4-3* is also able to cleave lycopene into bixin aldehyde, but at lower efficiency than *BoCCD1-1*, reflecting the catalytic differences between them.

## Discussion

The formation of bixin aldehyde from lycopene is considered as the first committed step of bixin biosynthesis (Fig. 1). This reaction involves the oxidative symmetric cleavage of lycopene at the 5,6/5′,6′ position to render bixin aldehyde. Although the isolation of a specific lycopene cleavage dioxygenase that catalyzes this reaction has been identified in an African *B. orellana* genotype (Bouvier, Dogbo & Camara, 2003), it has not been detected in deep coverage seed transcriptomes from Mexican cultivars (Cárdenas-Conejo et al., 2015). However, eight BoLCD-related sequences were found in a recent transcriptomic analysis of the Mexican NP4 accession (from the Yucatan Peninsula; Cárdenas-Conejo et al., 2015). These sequences were grouped into two classes: type 1 and type 4 CCDs (BoCCD1 and BoCCD4, respectively), which contained four members each (Fig. 3). Their possible involvement in bixin biosynthesis, as LCD enzymes, was analyzed by following their expression profile and assaying their catalytic properties. Three of the eight candidates showed a strong correlation with the developmentally associated bixin accumulation in seeds (Pearson’s *r* > 0.7; Table 2), but only two of them displayed the correct cell expression profile in the aril BSCs; one BoCCD1 and one BoCCD4, namely *BoCCD1-1* and *BoCCD4-3* (Figs. 6D and 6E). *BoCCD1-1* and *BoCCD4-3* were selected for functional analysis and the corresponding ORFs were expressed in a lycopene-accumulating *E. coli* strain (pACCRTEIB). Three lines of evidence support the lycopene oxidative cleavage activity of the heterologusly expressed BoCCD1-1 and BoCCD4-3 proteins. First is the consumption of the endogenously produced lycopene, detected as the color fading in the transformed cultures, which did not occur in the cultures transformed with empty vector (Figs. 7A–7C). Second, the MS patterns showed the formation of a product at *m/z* 349, consistent with the molecular mass of bixin aldehyde (Bouvier, Dogbo & Camara, 2003), simultaneously with a decreasing of the lycopene signal at *m/z* 536 (Figs. 7 and 8). Moreover, the expected aldehyde and keto groups were also detected in extracts from the recombinant cells (Fig. 9). Finally, cell-free extracts from the recombinant bacteria readily transformed externally added lycopene into the aldehyde, although at different efficiencies (Fig. 10; DOI 10.6084/m9.figshare.7967204). From these results, both *BoCCD1-1* and *BoCCD4-3* appear to correspond to lycopene cleavage dioxygenases. The previously reported BoLCD belongs to the CCD4 family and has been characterized to cleave lycopene in both a symmetrical (5,6/5′,6′) and nonsymmetrical (5,6 ó 5′,6′, 7,8 ó 7′,8′, 9,10 ó 9′,10′) fashions. The products of symmetrical cuts correspond to bixin aldehyde, whereas methyl 9′Z-apo-6′-lycopenoate or 6-methyl-5-hepten-2 are produced by the nonsymmetric cleavage (Lashbrooke et al., 2013; Rodrigo et al., 2013) (Fig. 1). Interestingly, the MS analysis revealed a *m/z* signal at 349, consistent with bixin aldehyde, but there were no signals corresponding to the alternative products 6-methyl-5-hepten-2-one (MHO) and pseudoionone.

On the other hand, although CCD1 and CCD4 enzymes can cleave C_40_-carotenoid and C_30_-apocarotenoides substrates (Lashbrooke et al., 2013; Schwartz, Qin & Zeevaart, 2001; Simkin et al., 2004), there is no previous report of a CCD-1 involved in bixin biosynthesis. Interestingly, most CCD1s cleave carotenoids at 5,6/5′,6′ positions, as does BoLCD (a CCD4 enzyme). Additionally, CCD1s can also cleave either end of the molecule to produce different compounds, such as MHO or other volatile compounds (i.e., pseudoionone) (Vogel et al., 2008). This suggests that CCD1 may also be involved in other processes, in addition to bixin biosynthesis. The differences in aldehyde production efficiency seem to support this interpretation. Genetic redundancy for the same catalytic activity is a common feature in plant secondary metabolism (Pichersky & Gang, 2000).

## Conclusions

In conclusion, we found two candidate genes, BoCCD1-1 and BoCCD4-3, which appear to have the same in vitro lycopene cleavage activity, although they are located in different cell compartments. Our data suggest that both classes of enzymes may be responsible for carotenoids metabolism. The primary objective of this work was to characterize and identify the BoCCDs involved in the initial step of bixin biosynthesis, but the subsequent steps and the mechanisms by which these enzymes take part in bixin biosynthesis remain uncertain. Therefore, we propose three possible mechanisms by which BoCCD4-3 and BoCCD1-1 are involved in bixin biosynthesis.

### Model for bixin biosynthesis

**Figure 11:**
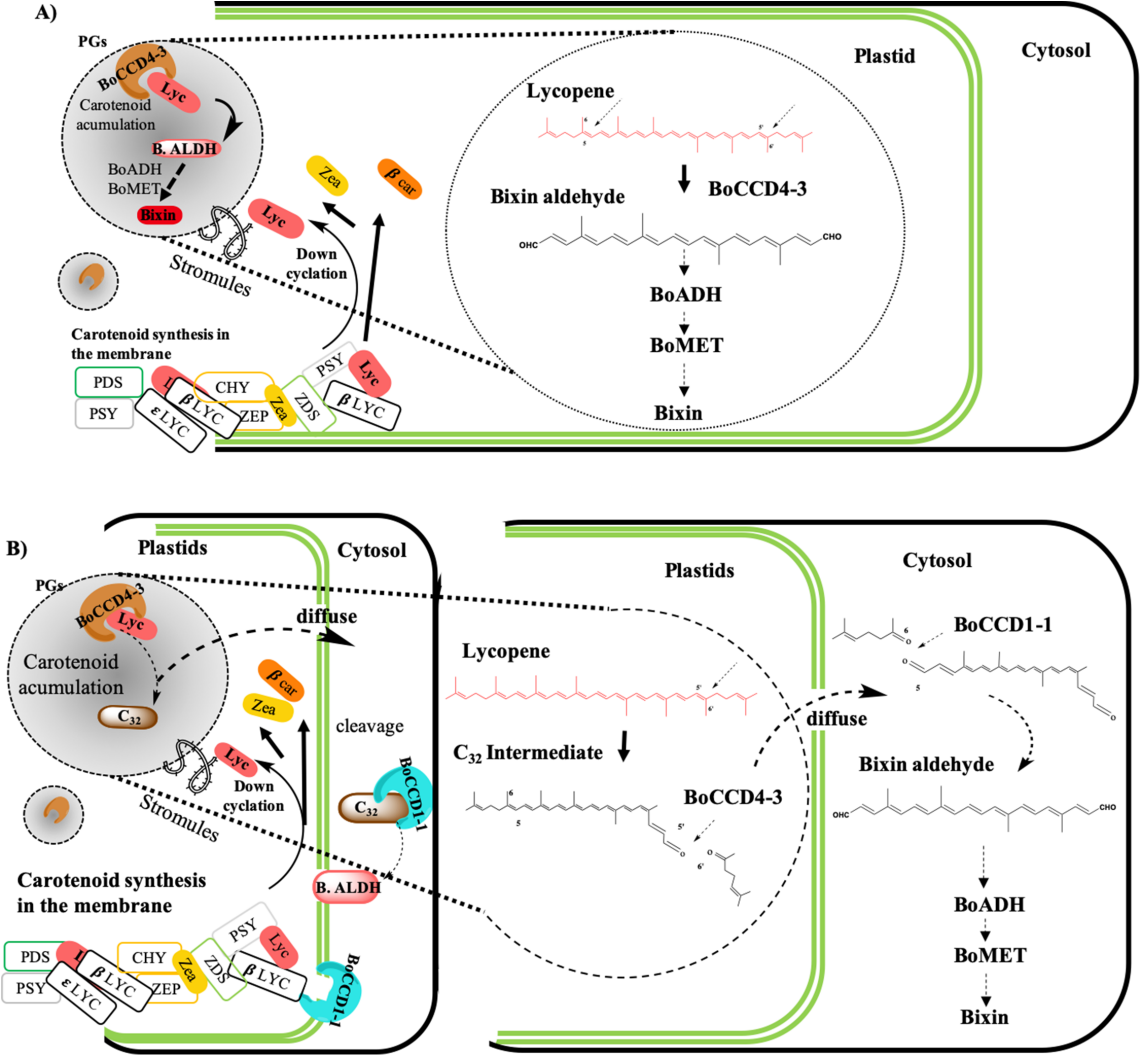
Bixin biosynthesis model of gene regulation in bixin biosynthesis. The black solid line represents the cell wall. The double green line represents the plastid. The grey circles represent plastoglobules (PGs). The structure in the form of “S” represent plastid stromules. Membrane-associated plastid complex enzymes: PSY, phytoene synthase; PDS, phytoene desaturase; ZDS, ζ-carotene desaturase; β-LCY, lycopene β-cyclase; ε-LCY, lycopene ε-cyclase; LYC, lycopene; β-Car, β-carotene; Zea, Zeaxanthin, B. ALDH., Bixin aldehyde; C_32_ (C_32_ intermediate). Bixin synthesis: BoCCD, Carotenoid cleavage dioxygenases; BoALDH, bixin aldehyde dioxygenase; BoMET norbixin methyltransferase (A) Bixin synthesis, is carried out in plastids: BoCCD4-3 cleaves lycopene symmetrically at position 5,6 (5′,6′) to produce bixin aldehyde and continues to bixin synthesis. Bixin could be transported by plastid stromules into BSCs. (B) Bixin synthesis is partially carried out in plastids and is finished in the cytosol. First BoCCD4-3 cleaves lycopene asymmetrically at position 5,6 or (5′,6′) and the resulting product (C_32_) diffuses out of the plastid and is used by BoCCD1-1 to form bixin aldehyde.

According to the findings of this investigation and considering the existence of the membrane-associated plastid complex and its role in the carotenoid synthesis, as well as the location of the carotenoid enzyme (Fraser, Schuch & Bramley, 2000; Joyard et al., 2009; Lopez et al., 2008; Sankari et al., 2018) (Fig. 11) we propose the following hypothetical models:

(**A**) Bixin synthesis is carried out in plastids, and BoCCD4-3 cleaves lycopene symmetrically at the 5,6 (5′,6′) position to produce bixin aldehyde. This hypothesis is supported by the differential expression of *BoCCD4* in the two achiote accessions, its correlation with bixin synthesis, and its location in plastids (Bréhelin, Kessler & Van Wijk, 2007; Kessler, Schnell & Blobel, 1999; Rey et al., 2000). In addition, *E. coli* protein expression seems to produce a compound with *m/z* 348.3, corresponding to bixin aldehyde (Bouvier, Dogbo & Camara, 2003). The CCD4 family has been reported to cleave lycopene both symmetrically and nonsymmetrically, forming bixin aldehyde via symmetric cleavage and methyl 9′Z-apo-6′-lycopenoate or 6-methyl-5-hepten-2 via nonsymmetric cleavage (Lashbrooke et al., 2013; Rodrigo et al., 2013) (Fig. 1). Moreover, the *E. coli* expression results and of the products analyzed by FTIR suggest that the compound is bixin aldehyde; in addition, lycopene was decreased as compared to the controls. Once bixin synthesis ends, it could be transported by plastid stromules into BSCs (Fig. 11A).
(**B**) Bixin synthesis is partially carried out in plastids and finished in the cytosol. If CCD4-3 cleaves lycopene at position 5,6, then its intermediate product (C_32_), could be exported into the cytosol by diffusion (Ahrazem et al., 2016). Once in the cytosol, BoCCD1-1 could use C_32_ as substrate and perform a second cleavage at position 5′,6′ to form bixin aldehyde (Fig. 11B). This hypothesis is supported by the observation that BoCCD1-1 does not have a plastid signal peptide and therefore, should remain in the cytoplasm without gaining access to lycopene; however, it could cleave the intermediate substrate (C_32_) outside the plastid. Additionally, this second hypothesis could be supported by considering the presence of the α-helical domain of the CCD1-1 enzyme (Fig. 3B), which could interact with the carotenoid enzymes associated with the plastid membrane (Kloer & Schulz, 2006; Messing et al., 2010). This mechanism has also been reported in roots of *Medicago truncatula* by Floss et al. (2008).
(**C**) Bixin synthesis is carried out in plastids and in the cytosol. Our results also suggest this third hypothesis, where both mechanisms **(A)** and **(B)** could occur simultaneously in the plant to synthesize bixin. Once bixin synthesis has ceased in the plastids or cytoplasm, it would be transported into the vacuole in carotenoid storage cells (Krieger et al., 2009; Louro & Santiago, 2016). As has been observed for other carotenoids originating in plastids, its final storage compartments are vacuoles and intercellular spaces (Bouvier et al., 2005; Camara & Bouvier, 2004; Giuliano, Rosati & Bramley, 2003; Gómez-Gómez et al., 2017; Grilli Caiola & Canini, 2004).

## Additional Information and Declarations

### Data Availability

The following information was supplied regarding data availability: The raw data is available at figshare: Carballo, Victor (2019): Dataset1.xlsx. figshare. Dataset. DOI 10.6084/m9.figshare.8127308.v1 Carballo, Victor (2019): Dataset2.zip. figshare. Dataset. DOI 10.6084/m9.figshare.8121224.v1 Carballo, Victor (2019): Dataset3.zip. figshare. Dataset. DOI 10.6084/m9.figshare.8121242.v1 Carballo, Victor (2019): Dataset4.zip. figshare. Dataset. DOI 10.6084/m9.figshare.8121212.v1 Carballo-Uicab, Victor Manuel (2019): Dataset5.zip. figshare. Dataset. DOI 10.6084/m9.figshare.7967198.v1 Carballo, Victor (2019): Dataset6.zip. figshare. Dataset. DOI 10.6084/m9.figshare.8171669.v1 Carballo-Uicab, Victor Manuel (2019): Dataset7.zip. figshare. Dataset. DOI 10.6084/m9.figshare.7967201.v1 Carballo-Uicab, Victor Manuel (2019): Dataset8.zip. figshare. Dataset. DOI 10.6084/m9.figshare.7967204.v1
